# A needs assessment for simulation-based training of emergency medical providers in Nebraska, USA

**DOI:** 10.1186/s41077-018-0081-6

**Published:** 2018-11-23

**Authors:** Nizar K. Wehbi, Rajvi Wani, Yangyuna Yang, Fernando Wilson, Sharon Medcalf, Brian Monaghan, Jennifer Adams, Paul Paulman

**Affiliations:** 1 Department of Health Services Research and Administration, College of Public Health, University of Nebraska Medical Center, 984350 Nebraska Medical Center, Omaha, NE 68198-4350 USA; 2 Department of Epidemiology, University of Nebraska Medical Center, Omaha, NE 68198 USA; 3 Department of Family Medicine, University of Nebraska Medical Center, Omaha, NE 68198 USA

**Keywords:** Emergency medical services, Emergency medical technicians, Needs assessment, Simulation training, Rural health

## Abstract

**Background:**

Training emergency medical services (EMS) workforce is challenging in rural and remote settings. Moreover, critical access hospitals (CAHs) struggle to ensure continuing medical education for their emergency department (ED) staff. This project collected information from EMS and ED providers across Nebraska to identify gaps in their skills, knowledge, and abilities and thus inform curriculum development for the mobile simulation-based training program.

**Methods:**

The needs assessment used a three-step process: (1) four facilitated focus group sessions were conducted in distinct geographical locations across Nebraska to identify participants’ perceived training gaps; (2) based on the findings from the focus group, a needs assessment survey was constructed and sent to all EMS and ED staff in Nebraska; and (3) 1395 surveys were completed and analyzed.

**Results:**

Thematic areas of training gaps included cardiopulmonary conditions, diabetes management, mass casualty incidents (MCI), maternal health and child delivery, patient assessment, pediatric care (PC), and respiratory emergency care. Gaps in non-clinical skills were related to crisis management such as maintaining effective teamwork. Participants frequently identified cardiopulmonary care, PC, and MCI as highly needed trainings. Other needs included life support-related retaining courses, sessions informing protocol updates, the availability of retraining tailored for rural areas, substance use-related emergencies, and farming-related injuries.

**Conclusion:**

EMS and ED staff identified several skill gaps and training needs in the provision of emergency services in rural communities. These results allow for the development of customized training curricula and, with the help of an on-site simulation-based program, can identify gaps in health professionals’ skills, knowledge, and abilities and thus help them respond to acute healthcare needs of rural communities.

## Background

One third of rural counties in Nebraska are either partially or wholly designated as a primary care health professional shortage area which are geographic areas or populations with too few primary care providers [1]. The poor access to care in rural communities highlights the importance of having an effective acute care infrastructure, consisting of regional emergency medical services (EMS) and critical access hospitals (CAHs) [2]. In 2016, Nebraska’s Department of Health and Human Services reported that there are 421 licensed emergency ambulance services, including 324 that provided basic services and are usually staffed with 1 or 2 emergency medical technicians (EMTs). Ninety-seven ambulance services were advanced life support ambulances [3]. Eighty-two percent of Nebraska’s EMS providers are volunteers who serve sparsely populated geographically large areas and have very limited resources [3]. Although EMS providers receive training and certification to provide emergency care, those trainings may be outdated or may not have provided sufficient and/or individualized training to help meet the healthcare needs in rural communities.

The number of EMS providers in rural Nebraska has been declining. From December 2013 to June 2015, the number of Nebraska licensed EMS providers dropped by 13% from 8436 to 7367. In the same period, the number of licensed EMS ambulance services dropped by 3% from 426 to 414 [4]. The number of state-licensed EMS instructors also fell by 14% (311 to 266). Many EMS agencies have small numbers of personnel. For example, of 414 EMS agencies, 170 (41%) have 10 or fewer licensed providers, and 35 (8%) have 5 or fewer [4]. Staffing in rural CAHs is highly variable that some of these facilities are staffed by residency-trained emergency medicine practitioners, while others are staffed by family practitioners. These staffing issues result in significant variation in team coordination and effectiveness across CAHs in delivering emergency medical care [4].

For many EMS volunteers and emergency department (ED) staff serving in rural areas, it is very challenging to find close-by training opportunities that will allow them to stay up-to-date in their skills. Moreover, volunteers have the burden of added cost of travel to where training is provided. Training is especially critical since highly acute but low-frequency situations in rural areas make it difficult for providers to practice emergency clinical skills.

To help address these training needs, the Simulation in Motion-Nebraska (SIM-NE) program was funded to provide a mobile, state-of-the-art simulation-based training program to EMS providers who serve in rural areas of Nebraska. SIM-NE consists of four purpose-built trucks each of which has two simulation labs; one simulates an ambulance scene and the other simulates an emergency room. The trucks are equipped with a variety of clinical instruments, placebo-based medications with prescription labels (e.g., injectable, antihypertensive), and high-fidelity manikins that simulate a wide range of health issues resulting from chronic and communicable diseases, pregnancy, and external causes of injury. Several studies showed that using high-fidelity simulation is an effective teaching tool because it increases participants’ learning satisfaction, self-confidence, performance, and self-efficacy [5–9]. Moreover, the use of such manikins and high-fidelity simulation provides educators with a more objective tool for participant evaluation and assessment of the training offered [9].

**Fig. 1:**
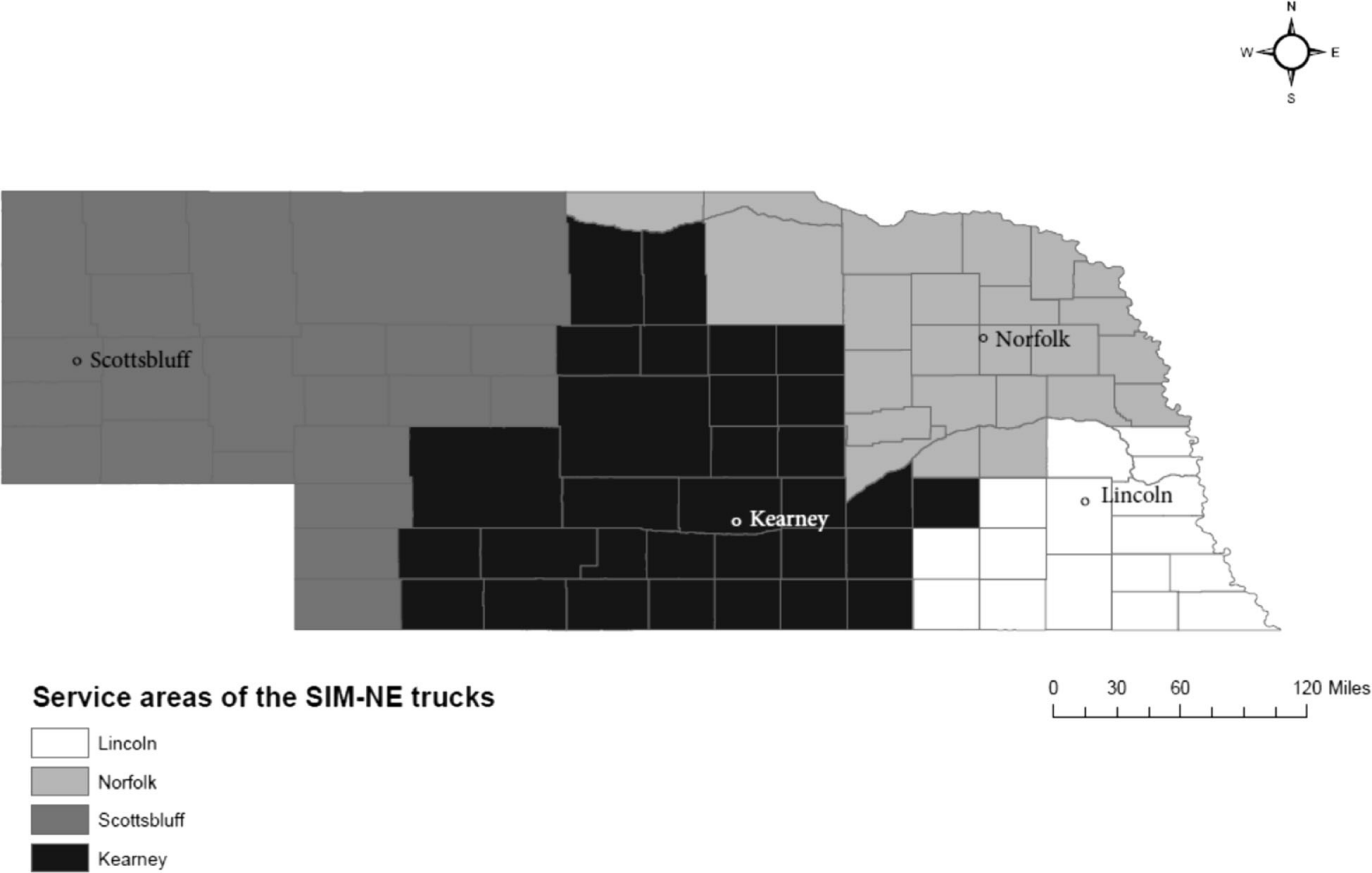
Map of Nebraska showing the simulation truck bases and service areas

Each of the four SIM-NE program trucks will be housed in a different city (Norfolk, Scottsbluff, Kearney, and Lincoln) in four distinct rural regions (see Fig. 1). Each truck will then be able to travel into more rural areas in the designated region and offer on-site training to EMS volunteers and ED staff. The SIM-NE program offers a valuable opportunity for emergency medical professionals to perform hands-on, realistic clinical activities that fall under their scope of practice in an informed, encouraging, and supportive learning environment [10].

In addition to the potential benefit of the SIM-NE program in offering on-site training to address gaps in rural Nebraska, it is possible to offer customized training modules based on the needs of trainees. Thus, we undertook a systematic, state-wide survey and analysis of EMS providers to identify specific gaps in skills and knowledge. A combination of qualitative analysis of focus group findings and a survey of all EMS providers licensed in Nebraska was used.

## Methods

This study followed a three-step process. First, we conducted focus groups in Nebraska at four distinct locations (Norfolk, Scottsbluff, Kearney, and Lincoln, see Fig. 1) to identify the thematic areas where the participants perceive there are gaps in their skills, knowledge, and abilities. Second, we developed a comprehensive survey tool and delivered to a large sample of healthcare professionals targeted by the SIM-NE program. Lastly, we analyzed the needs assessment survey; the results of which will inform the development of new and customized training modules in the future.

This study was deemed as a needs assessment/quality improvement by the Institutional Review Board at the University of Nebraska Medical Center (UNMC).

### Focus groups

The four focus group locations were selected based on geographical catchment areas and to mirror the four towns where the trucks will be housed from their service areas. The research team conducted the focus group sessions between November and December of 2016. Table 1 represents the distribution of participants of the focus groups by location. Semi-structured 90-min-long focus group sessions were conducted by a facilitator who facilitated the discussion with the following topics: (1) clinical skills with which you are less comfortable (or use infrequently) and would appreciate further training, (2) clinical skills for which you would like refresher training, and (3) non-clinical skills (e.g., patient report, teamwork, decision-making) for which you would like training (see Appendix). Using a collaborative consensus-building process, the facilitator guided the participants to cluster their responses in thematic groups. Through refining the data, it was possible to determine overarching areas that represent gaps in the participants’ collective skills, knowledge, and abilities.

**Table 1:** Distribution of focus group participants by location

Location in Nebraska	Number of participants working in ED	Number of participants working for EMS	Total number of participants
Lincoln	7	1	8
Norfolk	5	0	5
Kearney	10	3	13
Scottsbluff	8	0	8

### Needs assessment survey

The data collected during the focus groups identified 12 overarching thematic areas that were used to develop a survey questionnaire tool. The target population included physicians, physician assistants, advanced practice registered nurses (APRN), advanced EMTs, EMS instructors, EMTs, EMT-Intermediates, emergency medical responders, and paramedics. The Health Professionals Tracking Services (HPTS) at UNMC maintains data on the healthcare providers’ license type, age, gender, and practice locations. The HPTS provided the mailing address of the above target providers. A paper-based survey was mailed to 7676 licensed health professionals. The survey collected education attainment and practice setting from respondents as well as training needs, based on the themes identified by the focus group sessions. The survey asked the respondents to rate their need for emergency training on a 5-point Likert scale ranging from “no need” to “very high” (1 to 5 points). Training needs were categorized into the following 12 thematic areas: cardiopulmonary events, diabetic management, mass casualty incident, maternal health and child delivery, patient assessment, pediatric and infant care, respiratory emergency, trauma, basic clinical skills, behavioral health, communication, and patient reporting between providers, teamwork, and decision-making. Qualitative data, from an open-ended question, were also collected to probe for other gaps beyond those listed in the survey.

Quantitative data were analyzed using STATA software, version 14.0 (Stata Corporation, College Station, TX). Qualitative analysis, based on grounded theory, was performed by two independent coders to identify the common themes using MAXQDA software (Berlin, Germany) [11]. Grounded theory was used because it includes the identification and integration of categories or themes to derive meaning from collected qualitative data. The guidelines on how to identify categories and links or relationships between themes are provided as well as an explanatory framework to elucidate the study at hand [12].

## Results

### Focus groups

Table 1 represents the location distribution of participants who attended the focus group. Kearney had the highest attendance of 13 participants comprising both ED and EMS professionals while Norfolk had the lowest attendance of only 5 ED professionals. A total of 8 ED and EMS professionals attended the focus group session at Lincoln while participants at Scottsbluff included only 8 ED professionals.

Table 2 lists the thematic areas defined by the collaboration process. Each theme contains a list of clinical or non-clinical skills identified as a training need. All four locations identified similar challenges. Maternal health and child delivery was a common theme identified at all locations with participants requesting training for clinical skills like postpartum hemorrhage care, precipitous and regular delivery, shoulder dystocia, and neonatal resuscitation. Participants at all locations mentioned a need for refresher trainings for cardiovascular emergencies including stroke, cardiac arrest, acute myocardial infarction, and cardioversion. Similarly, participants from all locations unanimously requested retraining for respiratory emergencies, for example, intubation, respiratory arrest, anaphylaxis, and intraosseous needle insertion. Participants identified training needs for frequently encountered cases such as trauma of chemical exposure, electricity and fire, farming, neurology injury, and gunshots. Moreover, participants at all locations expressed a need for training to detect and treat behavioral health-related emergencies. Pediatric and infant care, including trauma and performing emergency procedures on pediatric patients, was also deemed as a training need at all four locations. All participants expressed a need to train themselves to help patients manage diabetes using the glucometer and insulin pump.

**Table 2:** List of clinical and non-clinical skills the participants of focus group need further training in

Themes	Types of skills
Behavioral health	Acute psychiatric conditions Detoxification Detection and treatment for behavioral emergencies
Cardiac care	Acute myocardial infarction Stroke Cardioversion/pacing Cardiac arrest management
Communication and handoffs between providers	Team transition Scene dynamics
Diabetes management	Diabetic ketoacidosis Insulin pump Glucometer
Maternal health and child delivery	Postpartum hemorrhage Delivery: precipitous/regular Abruption Shoulder dystocia Neonatal resuscitation
Pediatric and infant care	Pediatric trauma Emergency procedures
Respiratory emergency	Intubation Respiratory arrest Anaphylaxis Needle decompression/chest tube Intraosseous placement
Teamwork and decision-making	Continuation of care Decision to transfer (whole team) Multi-victim trauma
Trauma	Burns from overexposure to chemicals, electricity, and fire Farming/ranching injuries Neurotrauma Hemorrhage gunshots

The themes are arranged in alphabetical order

Besides the clinical skills, all participants emphasized the need for training on non-clinical skills. For example, training is needed to improve communication and efficient handoffs between providers during team transition.

### Needs assessment survey

#### Quantitative analysis

Table 3 shows that the response rate to the survey was 18.1% (1390 of 7676 providers). The majority of the respondents were male (58.7%). The average age of all respondents was 50.5 years (SD 13.7). The results showed that 31.4% of the respondents had an associate’s degree while 27.4% had a high school degree or general education diploma (GED). The majority of respondents (80.5%) practiced in the setting of emergency medical services (EMS) while only 3.2% practice in the emergency department (ED) and 14.3% practiced in both settings. The results showed that 68.2% had only an EMT license while 2.0% had an EMT and an EMS instructor license. Also, 4.8% were physicians and 3.5% were physician assistants. A few of the advanced practice registered nurses (APRN) had a second license such as EMT and paramedics.

**Table 3:** Descriptive characteristics of respondents of the needs assessment survey

Characteristics of respondents	Number (*N* = 1384)***	Percent
Age in years		
20–35	250	18.1
36–45	304	22.0
46–65	684	49.4
More than 65	146	10.5
Sex*		
Female	569	41.1
Male	812	58.7
Unidentified	3	0.2
Education level**		
Less than high school	3	0.2
High school/GED	379	27.4
Associate’s degree	435	31.4
Bachelors	325	23.5
Master’s degree	135	9.8
Doctorate	88	6.4
No response	19	1.4
Practice setting		
Emergency department	44	3.2
Emergency medical services	1114	80.5
Practice in both	198	14.3
No response	28	2.0
License type		
EMT/EMS		
Advanced EMT only	2	0.1
EMT only	944	68.2
EMT-Intermediate only	10	0.7
Emergency medical responder only	74	5.3
EMT + EMS instructor	27	2.0
EMT-Intermediate + EMS instructor	1	0.1
EMS instructor + emergency medical responder	1	0.0
Medical professionals		
Advanced practice registered nurse (APRN) only	24	1.7
Physician only	66	4.8
Physician assistant (PA) only	49	3.5
PA + EMT	2	0.1
APRN + EMT	4	0.3
APRN + paramedic	1	0.1
Paramedics		
Paramedic only	140	10.1
Paramedic + EMS instructor	39	2.8

*Gender information was provided by HPTS; gender information of three respondents showed unidentified in the HPTS database

**Education level and practice setting were collected from surveys. Few respondents did not answer these two questions

***Although we receieved 1390 surveys, there were 6 duplicates that were eliminated

Surveys asked the participants to rate their training needs in identified categories on a Likert scale from 1 to 5 (1, no need; 2, low; 3, moderate; 4, high; 5, very high). Table 4 categorizes 1 and 2 as low need and 3, 4, and 5 as moderate to high need. The majority of respondents (89.6%) rated “mass casualty incident” as moderate to high need, and 88.0% and 87.7% respondents rated “pediatric and infant care” and “respiratory emergency” as moderate to high need, respectively. While most respondents (84.4%) from Lincoln rated training of pediatric and infant care was moderately to highly needed, most respondents from Kearney (91.4%), Norfolk (90.3%), and Scottsbluff (90.3%) rated training of respiratory emergency care as moderate to high need.

**Table 4:** Frequency of training needs by skills and mean training needs rated by area

Skills	Moderate to high need* (%)	Moderate to high training need by area (%)			
		Lincoln	Kearney	Norfolk	Scottsbluff
Basic clinical skills	61.0	52.0	65.3	66.6	64.6
Behavioral health	70.5	62.0	74.7	71.7	86.6
Cardiopulmonary	85.3	82.3	85.5	87.3	90.0
Communication and handoffs between providers	60.0	55.0	61.7	64.0	64.6
Diabetic management	72.0	63.2	78.1	75.5	74.3
Mass casualty incident	89.6	87.6	87.9	93.5	92.0
Maternal health and child delivery	74.9	76.6	72.8	73.3	78.8
Patient assessment	70.5	64.4	74.2	75.1	69.9
Pediatric and infant care	88.0	84.4	90.6	89.9	88.5
Respiratory emergency	87.7	82.1	91.4	90.3	90.3
Teamwork and decision-making	65.0	58.5	70.4	68.2	66.4
Trauma	87.5	83.3	90.3	90.0	88.4

Surveys asked the participants to rate the training needs on a scale of 1 to 5. Training needs of 1 and 2 are categorized as low need and 3, 4, and 5 as moderate to high need

*The sum of individual counts may not add up to the total number of respondents because of missing information for certain variables

#### Qualitative analysis

We analyzed the responses obtained from the open-ended question “What other training do you think you need?” (Table 5). The results showed that 69% of the respondents did not indicate a need for any additional training. The nine key themes deemed necessary for training besides the ones proposed in the survey included (a) retraining of life support-related courses, (b) debriefing of protocols, (c) conducting rescue operations from fire- and water-based emergencies, (d) trauma, (e) substance use-related emergencies, (f) farming- and agriculture-related injuries, (g) administering medications and adverse drug reactions, (h) intubation and breathing difficulties, and (i) training tailored for rural areas. Based on the frequency of the responses, we ranked these themes and selected supporting quotes as shown in Table 5.

**Table 5:** Key themes, their frequency, and selected quotes from the responses to the needs assessment survey

Themes	Description	Frequency (%)	Selected quotes from survey responses
Retraining of life support-related courses	A need for refresher courses on EVOC, courses such as ATLS, PHTLS, AMLS, ACLS, and PALS and hands-on/practical training	133 (29.8)	“…need EMT courses, PHTLS, AMLS, ACLS, PALS. No NE Nebraska Regional Coverage for these courses [are provided] at regular intervals. [We] only get 1 shot per year, [which is] not conducive to unit member’s schedules...”
Debriefing of protocols	Further education on protocols, paperwork, record keeping	62 (13.9)	“...Multi agencies protocols i.e. hospital, and critical access’ protocols…”
Conducting rescue operations from fire- and water-based emergencies	Training on conducting rescue operations due to fire- and water-based emergency events	57 (12.8)	“...Any type of training that is geared towards fire and rescue...” and “…more information on search and rescue (at least training per year) ...”
Trauma	Training on helping during traumatic injuries	54 (12.1)	“...Essentially: stabilizing trauma for transfer (car accidents, sport injuries, etc.); caring for patients in the middle of a heart attack or stroke (what can we do to keep them alive); Caring for patients who may not be getting enough oxygen to survive the 20 minute ride with us to get definitive care...”
Substance use-related emergencies	Training in helping patients suffering from drug or alcohol misuse	34 (7.6)	“... narcotic/illegal drugs- what symptoms, for what days, how to combat…”
Farming- and agriculture-related injuries	Training on emergency events arising from farming or agriculture-related injuries or damage	31 (6.9)	“...Extrication training for vehicles used for farming and Agriculture...”
Administering medications and adverse drug reactions	Training on administering Narcan (naloxone), insulin, and other complex drug delivery systems; drug-drug interaction-related and adverse drug reactions; pharmacology to be adapted during complex differential diagnoses	28 (6.3)	“...would like capabilities to do IV’s administer Narcan and insulin I did not become paramedic because I would have had to move to have enough work. I live in very rural area I carry oxygen and airway masks, AED, Kings airways etc. in my personal vehicle...”
Intubation and breathing difficulties	Training in performing intubation and management of airway/breathing difficulties	28 (6.3)	“...Procedural skills: I would like to have an avenue to practice intubation periodically, particularly the medication used in rapid sequence intubation…”
Training tailored for rural areas	Training tailored for rural areas	20 (4.5)	“...More training on rural areas- how to get patient out and long transport. We do not have an ambulance in our town- only 3 volunteer EMTs- closest ambulance is 13 miles away…”

The frequency is calculated per comment obtained to the question, “What other training do you think you need?” by respondents

There were 133 respondents who mentioned a need for a refresher of Emergency Vehicle Operator Course (EVOC), Advanced Trauma Life Support (ATLS), Pediatric Advanced Life Support (PALS), and cardiopulmonary resuscitation (CPR). Around 14% of respondents expressed a need for education related to paperwork, protocols from health agencies, and methods to keep records. Fire- and water-related rescue operations were common across the state, and 12% of respondents mentioned a need to procure training for such emergencies. Fifty-four respondents indicated a need for a refresher training on stabilizing patients involved in trauma while 34 respondents requested education on treating patients who overdosed illegal drug and narcotics.

Thirty-one respondents requested training on caring for patients suffering from farming-related injuries and agriculture-related incidents. Administering medications such as naloxone, and helping patients combat adverse drug reactions, also requires hands-on training which respondents (28) believe can be provided by simulation-based education.

## Discussion

In this study, we undertook a comprehensive review of current training needs in rural Nebraska, USA, utilizing both in-depth focus group discussions and a broad-based quantitative survey. Results from this needs assessment identified key opportunities to improve the knowledge and skills among the workforce. These areas included treatment of cardiopulmonary conditions, diabetic management, mass casualty incidents, maternal health and child delivery, patient assessment, pediatric and infant care, and respiratory emergency care. Gaps in non-clinical skills were related to crisis management such as maintaining effective communication and teamwork among the EMS and ED providers. These findings will be used to develop curricula for an innovative mobile simulation program to address the rural community needs.

The provision of emergency services in rural areas involves unique challenges compared to urban settings. Such challenges include long travel distances, difficulties in assembling qualified teams, and agricultural-related injuries. Consistent with the study by Fleischman, responses from our study highlighted that ED and EMS personnel have few prior training sessions, infrequent incidences to apply clinical skills, and a generally low level of comfort in addressing emergencies [13]. Also, the prior literature suggests that providers in rural hospitals experienced inferior quality of education, lower number of practitioners, and limited financial resources compared to urban providers [2, 13]. Our findings present a clear need for a regular and updated training program tailored to the needs of rural EMS providers.

Infants and children account for approximately 4 to 13% of medical emergencies in rural areas [13, 14]. However, the standards mandated regarding the number of hours of pediatric training are inconsistent across different organizations such as the National Standard Curriculum, the National EMS Education Standard, and the National Registry of Emergency Medical Technicians [13]. This may explain the discomfort and limited experience with pediatric and infant care reported by our participants. The Institute of Medicine (IOM) reports that having a low volume of pediatric patients results in lower levels of preparedness for pediatric ED patients by rural emergency medical providers [15]. Previous literature has established the importance of conducting medical training focused on pediatric and infant emergency care [13, 16]. In fact, the SIM-NE program uses highly realistic manikins of newborns and infant children and thus will help address the concerns of our study participants regarding training in life-saving and complicated clinical procedures for pediatric patients. Such situations include obstetric hemorrhage and other delivery complications, neonatal resuscitation, intubation, and drowning. These concerns are likely to be shared by EMS providers in other rural communities.

Participants of our focus groups and respondents to the survey generally mentioned their lack of practical skills while treating trauma patients. The participants gave examples of multiple trauma activities for which they felt ill-equipped; such cases include burns (from chemical, electrical, and fire), head injuries, wounds from gunshots, amputations, profuse bleeding hemorrhage, and splinting. While an advanced trauma life support (ATLS) course typically uses simulated trauma scenarios, these courses are only available in post-graduate educational programs [17, 18]. However, a large proportion of EMS providers practicing in Nebraska usually does not have post-graduate degrees. Thus, training programs are needed to address this gap.

Almost 90% of respondents reported a need for further training in mass casualty incidents (MCI). Prior research suggests that simulation-based training for MCI can effectively increase critical thinking capacity, functionality within the healthcare team, and ability to learn from mistakes [19]. Effective handling of MCI requires a systematic evaluation of emergency preparedness and disaster awareness. Unfortunately, we found that rural EMS providers have few opportunities to develop skills in MCI within realistic scenarios. Our findings call for hands-on training programs to address readiness for manmade or natural disasters.

Our findings have informed curriculum development for the SIM-NE trainings that will specifically address the skills and knowledge gaps identified by our focus groups and survey results. For example, a considerable proportion of responses emphasized the importance of glucometers and insulin pumps to aid emergency treatment of diabetes patients. To address this concern, the SIM-NE truck has the capability to train EMS providers in treating acute cases of uncontrolled diabetes. By grounding training programs to the needs assessment of rural communities, simulation-based programs can provide training that is responsive to these needs.

Our findings also identified several non-clinical areas of concern in emergency services, such as teamwork. Although successful team leadership as well as safe and effective delivery of prehospital care is required as part of the National EMS Education Standards, the components of effective teamwork have not been clearly defined [20, 21]. Consistent with previous studies [20, 22, 23], the important characteristics of effective teamwork that the respondents rated as “high need” for training include situational awareness, task management, coordinating dissimilar information, decision-making, and communication. Having both a simulated ED and ambulance unit within the SIM-NE truck, it is possible to evaluate and enhance teamwork for both EMT and ED staff. Other important issues impacting rural healthcare identified through our focus group discussions are significantly limited resources among rural EMS providers, small emergency care teams, and insufficient financial support for hands-on training.

It is very important to realize that our study findings are as beneficial to other rural communities and sparsely populated areas in the USA or even in the world. Such challenges facing ED and EMS providers are many times similar by the virtue of travel distances, limited resources, and low frequency of emergent cases.

This study has limitations. Organizing the focus groups included challenges such as low rates of participation by ED and EMS professionals. The unequal representation of ED and EMS providers and the small representative sample of the focus groups might have resulted in missing some of the training needs. Moreover, ED and EMS providers although react to similar emergency cases, each has a different set of required skills and competencies. Having a more representation of each group would have allowed us to capture more of the nuances that exist. Nonetheless, the focus group process was meant to provide the foundation for survey development.

The survey was not checked for validity and reliability or piloting. It might be argued that individuals might interpret questions in different ways. Nonetheless, the research team was ensured that the survey questions were simple and straightforward to minimize or even eliminate any variation in interpretation. Moreover, the intent of the study was to identify those training gaps and needs that would allow the SIM-NE program to better address these gaps.

Although we mailed the survey to 7676 licensed health professionals, we had a low response rate of 18%. This might be a result of the fact that the majority of the EMS providers are volunteers and responding to surveys might not be top-of-mind. One can always argue that it might not be a representative sample or that it might suffer from selection bias. Nonetheless, we received 1390 responses which are a sizable cohort for our study and provided a rich dataset for analysis.

## Conclusions

Few prior studies have undertaken a needs assessment of emergency services providers in predominantly rural states. In Nebraska, many EMT providers are volunteers and have limited access to resources, experience in applications of skills, and training opportunities to maintain or improve their clinical and non-clinical skills. Similarly, many rural ED staff may not receive many opportunities to address complex or challenging cases requiring emergent care. The inferences from focus group discussions and survey highlight a clear need for clinical and non-clinical training to ensure the effectiveness of the emergency services workforce. The findings of our study are not just limited to rural Nebraska but might also be applicable to other rural areas in the USA as well as the world. These results provided the basis to develop customized training curricula for EMT and ED staff in rural areas delivered through an on-site state-of-the-art mobile simulation-based program. Such method of delivery might be a model for colleges and other training programs to build capacity and be responsive to ongoing and unique needs of the healthcare workforce in rural communities.

## Appendix

### Guide to facilitate focus group discussion

1. Make a list of clinical skills with which you are less comfortable (or use infrequently) and would appreciate further training
2. Make a list of clinical skills for which you would like refresher training
3. Make a list of soft skills (i.e., patient report, teamwork, decision-making) for which you would like training

## Abbreviations

AMLS: Advanced Medical Life Support
APRN: Advanced practice registered nurse
ATLS: Advanced Trauma Life Support
CAH: Critical access hospital
CPR: Cardiopulmonary resuscitation
ED: Emergency department
EMS: Emergency medical services
EMTs: Emergency medical technicians
EVOC: Emergency Vehicle Operator Course
GED: General education diploma
HPTS: Health Professionals Tracking Services
IOM: Institute of Medicine
MCI: Mass casualty incidents
PALS: Pediatric Advanced Life Support
PC: Pediatric care
PHTLS: Prehospital Trauma Life Support
SIM-NE: Simulation in Motion-Nebraska
UNMC: University of Nebraska Medical Center
